# Acute Exertional Compartment Syndrome in a Young Amateur Soccer Player: A Case Report

**DOI:** 10.5334/jbr-btr.904

**Published:** 2016-04-15

**Authors:** Julie Lambert, Ruth Ceulemans

**Affiliations:** 1 University Hospitals Leuven, BE

**Keywords:** Compartment syndromes, Ultrasonography, Magnetic Resonance Imaging

## Abstract

We present a case of acute compartment syndrome of the lateral compartment of the lower leg, caused by strenuous eccentric exercise. The diagnosis can be made based on clinical findings, but ultrasound and MRI can be useful in recognizing this rare form of compartment syndrome.

## Case History

A 21-year old male patient presented to the emergency department with pain in the lateral aspect of his left lower leg. The pain started shortly after playing a soccer game in competition. No trauma occurred during the game.

An ultrasound exam of the lower leg was ordered, which showed extensive subcutaneous edema. In combination with redness and elevated temperature of the overlying skin, the imaging findings were interpreted as infectious cellulitis. Over the next few days, all clinical findings and the pain increased, and the lateral lower leg became swollen. However, the patient’s overall temperature was normal, and he showed no signs of systemic illness.

**Figure 1:**
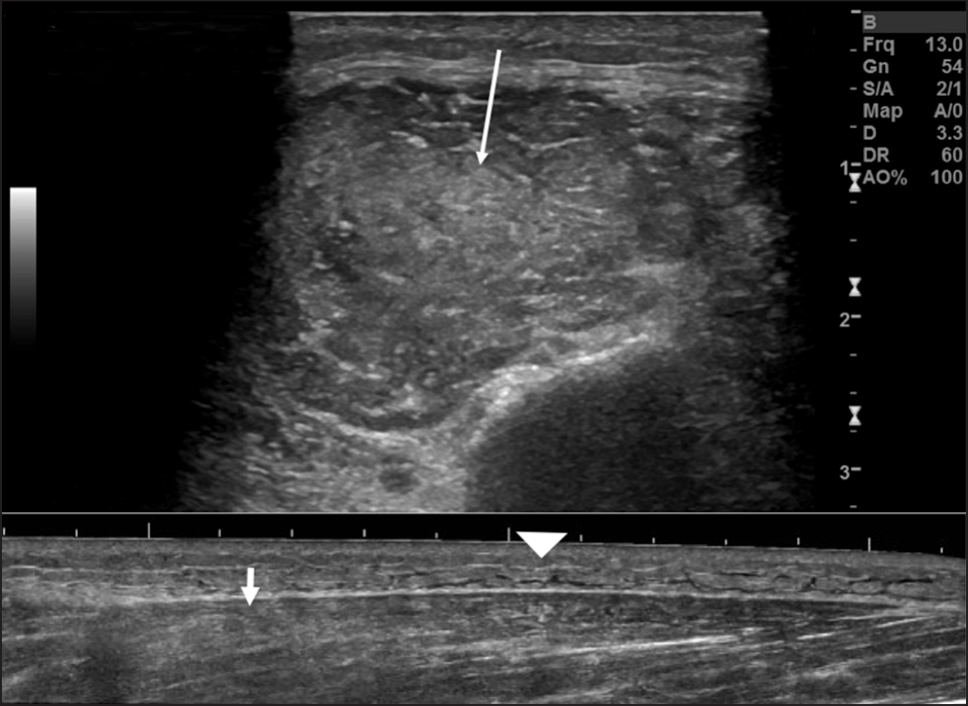
Ultrasound exam. *Top:* Transverse image of the peroneal muscle compartment shows increased echogenicity (long arrow), compatible with myositis. *Bottom:* Longitudinal image with muscle swelling (short arrow) and subcutaneous edema (arrowhead) in the proximal one-third of the lower leg.

He then developed a drop foot, and a second ultrasound exam was ordered to evaluate the common peroneal nerve. On imaging, the nerve demonstrated a normal caliber and echotexture, without extrinsic compression by a discrete mass or fluid collection. However, swelling and increased echogenicity of the peroneus longus and brevis muscles were apparent (Figure 1).

**Figure 2:**
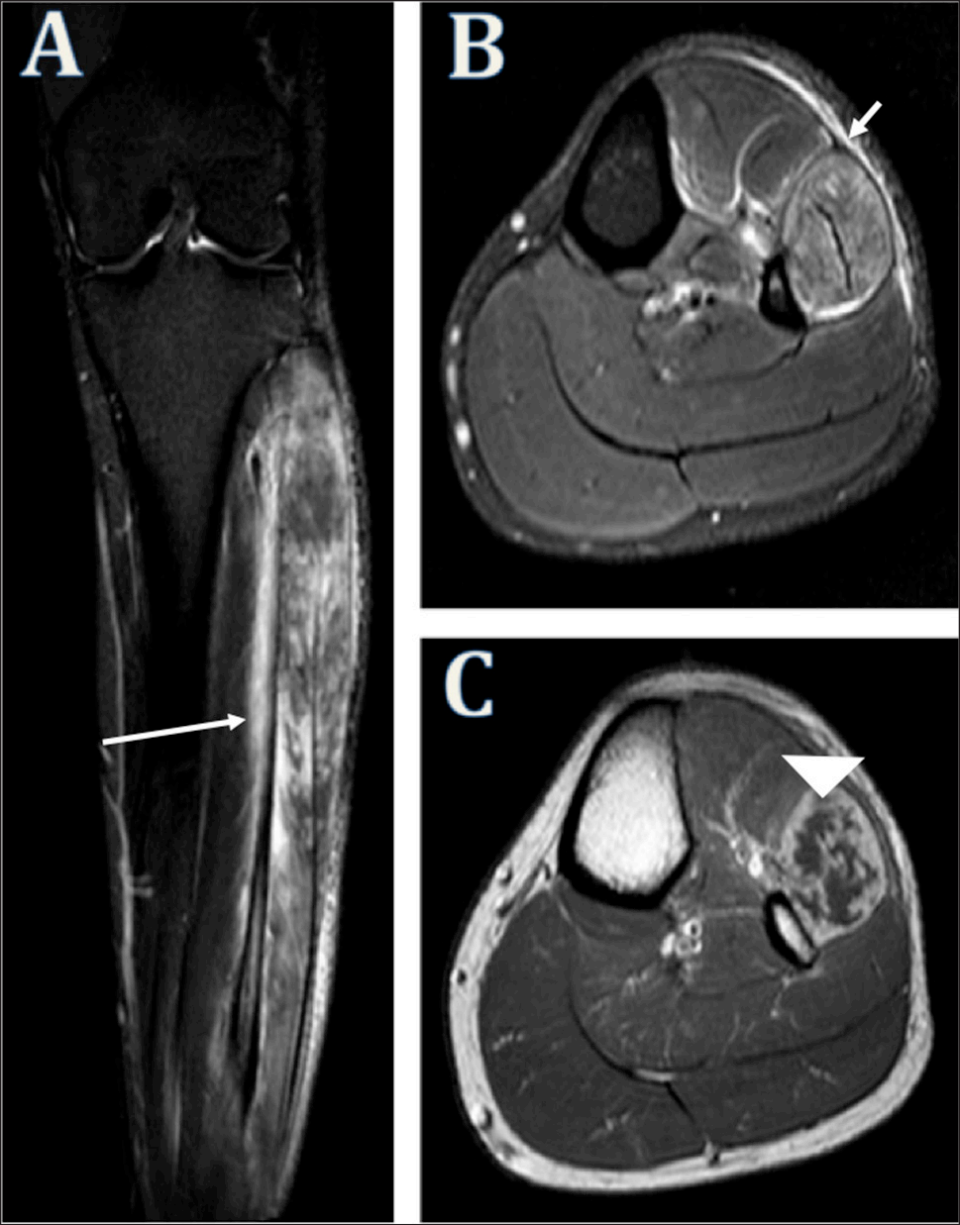
*A.* Coronal STIR image depicts diffuse high signal intensity edema in the peroneal muscle compartment (long arrow). *B.* Axial T2 SPAIR image shows globular morphology of peroneal muscle compartment with outward convex bulging of the overlying crural fascia, muscle edema, and subcutaneous edema along the fascial plane (short arrow). *C.* The axial contrast-enhanced SE T1-weighted image shows the nonenhancing, necrotic muscle tissue (arrowhead).

A subsequent magnetic resonance (MR) exam showed swelling and edema in the peroneal muscle compartment, most extensive in the proximal half of the leg (Figure 2). SE T1-weighted imaging following IV contrast administration demonstrated severe muscle necrosis. On T2-weighted imaging, adjacent edema in the deep subcutaneous fat along the fascia, in the fibular bone marrow, and in the distal course of the common peroneal nerve was also identified.

## Discussion

The lower leg consists of four compartments: an anterior (extensor), a lateral (peroneal), a superficial posterior, and a deep posterior (flexor) muscle compartment, each of them enclosed by a firm fascia and separated by either the interosseous membrane or an intermuscular septum. Rapid volume increase in a compartment inevitably results in increased pressure in this compartment. Compartment syndrome occurs when the interstitial pressure rises above the diastolic blood pressure, and as a result capillary perfusion is compromised [1].

Acute trauma is the most frequent cause of acute compartment syndrome. This etiology is well known and studied.

Compartment syndrome can also be caused by vigorous exercise and mostly manifests in a chronic form, with symptoms spontaneously resolving after rest [2]. The more rare, acute form of exertional compartment syndrome was first introduced as ‘march gangrene’ in 1945 by Vogt [2,6]. The pathophysiology of exertional compartment syndrome is known in the literature as the mechanical damage theory. It states that strenuous eccentric exercise and the resulting myofiber damage cause the release of protein-bound ions, which increase the osmotic pressure in the compartment. The fiber swelling and increased compartmental blood volume initiate a vicious cycle: the elevated compartmental pressure hampers muscle perfusion, and the edema secondary to the subsequent myoneural ischemia increases the compartmental pressure even further, eventually resulting in muscle necrosis [2].

Recognizing the clinical signs of compartment syndrome is crucial for diagnosis, regardless of the underlying cause. Pressure measurements can be additionally performed, with normal pressures ranging between 0 and 15 mmHg [3]. Two imaging studies can also be useful. First, although increased reflectivity and swelling of the muscles on ultrasound are nonspecific findings, they indicate muscle edema, as seen in compartment syndrome. Besides, it is also a fast and reliable test to exclude other causes of acute lower extremity pain [4,5]. Second, MR is an even better modality for soft-tissue evaluation, including muscle [6]. The T2-weighted signal intensity of muscle increases during exercise, due to the higher water content. MR has already proven to be useful in the setting of chronic exertional compartment syndrome. There is a statistically significant increase of T2-weighted signal intensity in the extensor compartment during exercise in patients with chronic compartment syndrome in comparison with the control group [7]. In acute compartment syndrome, muscle edema results in increased T2-signal intensity and, when hemorrhage is present, in increased SE T1 signal intensity. Following IV gadolinium administration, necrotic muscle tissue is depicted as a nonenhancing intramuscular region on SE T1-weighted sequences[8]. However, obtaining an MR exam can cause a delay and result in a poor outcome [2]. Therefore MR can only assist in the diagnostic workup of acute exertional compartment syndrome when it is rapidly available.

## Conclusion

Acute exertional compartment syndrome has to be included in the differential diagnosis of a painful and swollen leg. Clinical suspicion for this atraumatic cause of compartment syndrome is key, but ultrasound can be helpful in the acute setting. Above all, MR is a very sensitive imaging modality for confirmation of this diagnosis, with an important note being that obtaining it cannot delay the diagnostic process.

## Competing Interests

The authors declare that they have no competing interests.
